# Development of Green Fluorescent Protein-Tagged Strains of *Fusarium acuminatum* via PEG-Mediated Genetic Transformation

**DOI:** 10.3390/microorganisms12122427

**Published:** 2024-11-25

**Authors:** Fangyi Ju, Zhongqiang Qi, Jiajin Tan, Tingting Dai

**Affiliations:** 1 Co-Innovation Center for the Sustainable Forestry in Southern China, Nanjing Forestry University, Nanjing 210000, China; 15850535371@163.com (F.J.); tanjiajin@njfu.edu.cn (J.T.); 2 Institute of Plant Protection, Jiangsu Academy of Agricultural Science, Nanjing 210014, China; qizhongqiang2006@126.com

**Keywords:** *F. acuminatum*, transformation, PMT, GFP

## Abstract

*Fusarium acuminatum* is recognized as the causative agent of root rot in many forestry and agricultural plants. In recent years, root rot and foliage blight caused by *F. acuminatum* have become widespread and severe in China, particularly affecting *Dianthus chinensis*. The infection mechanism of *F. acuminatum* remains a pressing area for research. A crucial approach to elucidating its pathogenic mechanisms involves the genetic modification of candidate genes, which necessitates effective transformation systems. Currently, protoplast-mediated transformation (PMT) serves as a valuable tool for studying plant-pathogen interactions and offers several advantages over conventional transformation methods. In this study, we employed the PMT technique to establish a transformation system for the *F. acuminatum* strain FDCY-5 due to its benefits such as ease of operation, low cost, high conversion efficiency, and broad applicability. We successfully developed a transformation system capable of producing abundant high-quality protoplasts from *F. acuminatum* and generating green fluorescent protein (GFP) transformants. To verify whether GFP was constitutively expressed, we utilized fluorescence microscopy alongside PCR technology. The results demonstrated that GFP was effectively transformed into the protoplasts of *F. acuminatum* and expressed successfully. The established protoplast transformation system for *F. acuminatum* provides a foundational platform for analyzing functional genes within infected host plants as well as understanding the molecular mechanisms underlying host plant infections by *F. acuminatum.*

## 1. Introduction

*Fusarium* species are widely recognized for their prevalence in nature, with more than 400 distinct species identified [1]. Some of these species pose significant threats to the health of animals, insects, and plants. A considerable number of *Fusarium* spp. are capable of producing mycotoxins such as trichothecenes, zearalenone (ZEN), and fumonisins, along with other secondary metabolites that have been linked to symptoms including vomiting, oral lesions, and reproductive disorders in domestic animals [2]. Additionally, they exhibit entomopathogenic properties toward mealworms, flour beetles, maize borers, and blow flies [3]. *Fusarium* species, as plant pathogens, typically induce vascular system diseases in plants by infecting their roots. These fungi produce a chemically diverse array of mycotoxins and can lead to severe root rot, ultimately resulting in diminished yields and compromised quality and value of the plant products. This situation contributes to significant economic losses on a global scale [4]. *F. graminearum* is responsible for causing *Fusarium* head blight (FHB), one of the most devastating diseases affecting wheat and barley—two major crops globally. This plant disease poses a significant threat to grain production and leads to substantial reductions in yield [5]. Additionally, *F. oxysporum* is a soil-borne pathogenic fungus with a worldwide distribution that can infect the vascular bundle system of plants, resulting in wilting [6,7]. In the mid-20th century, *Fusarium* wilt caused by *F. oxysporum* severely impacted the banana trade and posed threats to various banana varieties across the globe [8]. 

*F. acuminatum,* a member of the *F. tricinctum* species complex (FTSC), has been reported to cause root rot and leaf spot [9]. Currently, plant diseases attributed to *F. acuminatum* are frequently observed in various countries, including eastern Australia [10], South Africa [11], USA [12] and China. *F. acuminatum*, like other species within the Fusarium genus, is capable of producing various mycotoxins, including deoxynivalenol (DON), T-2 toxin and HT-2 toxin. These mycotoxins are known to induce wilt and root rot in legumes [13,14] as well as in a range of crops including wheat [15], lentil [16], kiwifruit [17,18], *Ophiopogon japonicus* (Linn. f.) [19], *Dianthus chinensis* [20], *Astragalus membranaceus* [21], and *Atractylodes lancea* [22]. According to a recent report, this pathogen can even cause leaf spot on garlic [23]. *F. acuminatum* is widely distributed across the globe, and the cultivation of host plants for *F. acuminatum* may facilitate the rapid propagation and dissemination of plant diseases throughout significant regions of China. Therefore, there is an urgent need for reasonable and effective disease management strategies.

Currently, researchers commonly employ four distinct methods to obtain transformants from cells: gene gun bombardment, electroporation, Agrobacterium-mediated transformation (AMTM), and polyethylene glycol/calcium chloride-mediated protoplasts transformation (PMT) [24]. AMTM is extensively utilized in plant research [25], while both AMTM and PMT have gained significant traction in recent studies involving fungi. In comparison to AMTM, PMT offers a simpler and more effective approach that does not necessitate costly equipment [26]. The transformant system facilitates an in-depth investigation of gene function in fungi. Protoplasts are generated through enzymatic hydrolysis of cell walls, followed by polyethylene glycol (PEG) mediated transformation. Under the influence of PEG, exogenous DNA is introduced into the target cells, and transformants are subsequently obtained via resistance screening [27]. This approach enables researchers to specifically target and efficiently modify genes, thereby elucidating the functions of specific genes or incorporating exogenous genetic elements into the strain’s genome to alter the expression of endogenous genes [24]. This method was first established in *Saccharomyces cerevisiae* [28]. Currently, over a hundred filamentous fungi have been transformed using this technique, and a comprehensive transformation system has been developed [29]. In the field of plant pathology, this transformation system has found extensive application in studying gene function within pathogens and host plants, elucidating infection patterns of pathogens in host organisms and allowing for the investigation of interaction mechanisms between pathogenic fungi and their hosts through approaches such as CRISPR-Cas9 and homologous recombination [30,31,32]. For instance, research on *Magnaporthe grisea*, a destructive pathogen affecting rice, has utilized PEG-mediated protoplast transformation to uncover the functions and mechanisms of numerous genes and proteins that are critical to infection processes and pathogenicity [33]. Employing an appropriate transformation system, researchers employed green fluorescent protein (GFP) to analyze changes in infection patterns of *F. graminearum* with a disrupted trichodia synthase gene compared to its wild-type strain [34]. Yao et al. utilized GFP to investigate the interactions between the endophytic tomato fungus *Acremonium implicatum* and *Solanum lycopersicum* [35]. Additionally, Cai et al. employed confocal laser microscopy to observe colonization dynamics and distribution patterns of GFP-tagged *Metarhizium anisopliae* in maize [36].

Because the components of the cell wall are generally amenable to enzymatic hydrolysis by various cell wall enzymes, it is easy to obtain a large number of protoplasts. PEG, which can change the permeability of cell membranes, has frequently been employed for the introduction of exogenous DNA into protoplasts of various *Fusarium* species, including *F. graminearum* [37], *F. virguliforme*, *F. Brasiliense* [38], *F. oxysporum* [39,40,41], and *F. nematophilum* [29]. Utilizing PEG-mediated protoplast transformation techniques, researchers successfully introduced a knockout cassette into *F. graminearum*. And in subsequent experiments they revealed the potential involvement of the *FgPDE1* gene in conidia formation [42] and that the *PKS4* gene is essential for zearalenone production [43]. However, there have been no studies on protoplast transformation in *F. acuminatum*.

The objective of this study was to develop GFP-tagged strains of *Fusarium acuminatum* and an effective PEG/CaCl_2_-mediated protoplast transformation system for the further investigation of gene function in *F. acuminatum*. In this study, we established a highly efficient enzymatic hydrolysis system that enabled the successful collection of substantial quantities of protoplasts from *F. acuminatum* and successfully obtained GFP-tagged strains through an optimized transformation system. Furthermore, it was confirmed that the transformation system we developed did not adversely affect the growth or pathogenicity of the wild strain when foreign genes were introduced, thereby providing a reliable method for identifying genes related to pathogenicity. And GFP-tagged strains of *Fusarium acuminatum* can be used for the further observation of colonization dynamics and distribution patterns.

## 2. Materials and Methods

### 2.1. Strains, Culture Media and Plasmids

The *F. acuminatum* strains utilized in the present study were isolated from the roots of *Dianthus chinensis* samples collected at Nanjing. The samples were preserved on dried filter sheets at −20 °C in our laboratory. The FDCY-5 strain was subsequently cultured on complete medium (CM: 6 g NaNO_3_, 1.52 g KH_2_PO_4_, 0.52 g MgSO_4_•7H_2_O, 0.52 g NaCl, 1 mL trace elements, 1 mL vitamin solution, 10 g D-glucose, 2 g peptone, 1 g yeast extract, 1 g casamino acids, 15 g agar, add distilled water to 1 L) at 28 °C. Regeneration medium (RM: 1 g yeast extraction, 1 g casein enzymatic hydrolysate, 342 g sucrose, 16 g agar, add distilled water to 1 L) without antibiotics was used for regenerating the protoplasts and selection medium (SM: 1 g yeast extraction, 1 g casein enzymatic hydrolysate, 342 g sucrose, 10 g agarose, add distilled water to 1 L) supplemented with 60 μg/mL hygromycin B (Hyg) was used to screen HPH-resistant transformants. Mung bean medium (MBM: 15 g mung bean, add distilled water to 1 L) was used for culturing strains to produce conidia. Minimal medium (MM: 6 g NaNO_3_, 1.52 g KH_2_PO_4_, 0.52 g MgSO_4_•7H_2_O, 0.52 g NaCl, 1 mL trace elements, 1 mL vitamin solution, 10 g D-glucose, 15 g agar, add distilled water to 1 L), potato dextrose agar medium (PDA: 200 g potato, 20 g glucose, 15 g agar, add distilled water to 1 L) and 5 × YEG (10 g D-glucose, 5 g yeast extract, 20 g agar, add distilled water to 1 L) were used for growth observation of strains. The GFP expression vector (pKD1::GFP) employed in this study is a preserved plasmid from our laboratory that contains two expression cassettes: one encoding the visual reporter gene GFP and the other serving as the selectable marker gene conferring resistance to hygromycin B (Hyg) through hygromycin production.

### 2.2. Antibiotic Resistance Screening

To determine the most suitable concentration of Hyg for selection, the mycelium of FDCY-5 was inoculated onto CM medium containing varying concentrations of Hyg antibiotics: 0, 10, 20, 30, 40, 50, 60, 70, 80, and 90 μg/mL. The cultures were incubated at a temperature of 28 °C for five days to observe the growth patterns. The growth of each colony was monitored and compared in order to establish the minimum effective concentration of Hyg for the *F. acuminatum* isolate FDCY-5.

### 2.3. Preparation of Protoplast and Transformation

The mycelia of *F. acuminatum* of 3 days (agar plugs were not excluded) were cut into 25 pieces (2 × 2 mm) using a sterile scalpel and cultured in CM liquid at 28 °C with shaking at 160 rpm for 16–20 h to obtain sufficient fungal biomass. The mycelia were then filtered through sterile filter cloth and incubated in 20 mL of protoplast solution containing 0.2 g lysing enzymes from *Trichoderma harzianum* (Sigma Cat# 1412, SIGMA-ALDRICH, Co., Saint Louis, MO, USA), 0.15 g Driselase from *Basdiomycetes* sp. (Sigma Cat# D9515), 0.01 g chitinase from *Streptomyces griseus* (Sigma Cat# C6137) and 20 mL 0.7 M NaCl for 2 h, 70 rpm and 30 °C. To determine the optimal time for maximal protoplast yield, five different digestion times (90, 120, 150, 180, and 210 min) were evaluated to assess their impact on both the quantity and quality of protoplasts using a hemocytometer. This experiment was conducted in triplicate; the resulting data on protoplast quantities were analyzed via one-way ANOVA and Duncan’s range test in IBM SPSS Statistics 26. After digestion, the protoplasts were centrifuged at 2000 rpm and 4 °C for 5 min, and the supernatant was discarded. The collected protoplasts were then resuspended in 10 mL 0.7 M NaCl and centrifuged for 5 min, 2000 rpm, 4 °C. Next, 1 mL STC (145.744 g sorbitol, 6.057 g Tris, 5.5495 g CaCl_2_, add distilled water to 1 L) solution was added to each of the centrifuge tubes for resuspension purposes. The protoplasts were collected by centrifugation at 2000 rpm for 5 min and resuspended at 2–3 × 10^8^/mL in 1 mL STC solution, adding a quarter volume of SPTC (60% PEG4000 of STC) solution and then placed on ice for transformation. Then, 30 µg of plasmid was added to the 150 μL of protoplasts, and the mixture was gently mixed thoroughly before being placed on ice for 30 min. Subsequently, 1 mL of SPTC was incorporated into the mixture. After allowing it to stand at room temperature for 20 min, the mixture was transferred into RM at 43 °C. The protoplast and RM mixture were plated onto 9 mm disposable plastic Petri dishes and then incubated in the dark at 25 °C for 12 h. Following this incubation period in RM, SM was overlaid on the plate, which was then inverted and maintained at 25 °C for an additional four days.

### 2.4. DNA Extraction and Detection of Transformants

The genomic DNA was extracted from strains cultivated on CM medium plates for three days using the cetyltrimethyl ammonium bromide (CTAB) method. The concentration of the DNA extract was measured with a microspectrophotometer (BioPhotmeter^®^, Eppendorf Co., Hamburg, Germany), ensuring that the A260/A280 ratio remained optimally between 1.8 and 2.0.

The FDCY-5 transformants were carefully picked using toothpicks and subsequently placed on CM plates containing 60 μg/mL Hyg antibiotics. Following a culture period of three days, the mycelium and spores of the transformants were transferred onto a microscope slide, to which a drop of sterile water was added to facilitate unfolding. The mycelia and spores were examined at a wavelength of 488 nm using a fluorescence microscope (Zeiss Axioscope 5, Carl Zeiss AG Co., Oberkochen, Germany) to confirm the presence of fluorescence. To identify positive transformants, PCR was performed on each DNA template at a concentration of 100 ng/μL. The primers used for amplification were GFP-F (GACGACGGCAACTACAAG) and GFP-R (GAACTCCAGCAGGACCAT), designed to amplify a fragment length of 366 bp. The polymerase chain reactions conducted in this study were performed by using 50 μL reaction mixtures containing 25 μL of Green Taq Mix (Vazyme Biotech Co., Nanjing, China) 2 μL, Forward Primer 2 μL, Reverse Primer 2 μL, 200 ng template DNA, and 19 μL distilled water. And the PCR program was: 94 °C for 5 min, 33 cycles of 94 °C for 30 s, 56 °C for 15 s, and 72 °C for 30 s, and a final extension at 72 °C for 10 min. The products of PCR were visualized by 1.5% agarose gel electrophoresis. The genomic DNA of the wild strain (WT) and ddH_2_O (CK) as the negative control and vector containing the GFP gene as the positive control were used as controls for the reaction. The experiment was repeated three times.

### 2.5. Biological Determination of GFP Labeled Strain GFP-1

We selected a representative transformant (FcGFP-1) as the experimental material for subsequent experiments. The mycelial plugs of the wild strain and positive transformant with same size for 3 days of growth were prepared to observe the colony morphology and the growth rate. The mycelium plugs were cultured on Petri dishes containing different media including CM, MM, PDA and 5 × YEG for 5 days of growth. After culturing at 28 °C for 5 days in a dark incubator, the diameters of the fungal colonies were measured by using a cross method with mycelium plugs as the center, using three independent replicates and calculating the average value. Six blocks of mycelium plugs were cultured in CM liquid at 28 °C 160 rpm for 16 h of growth. The mycelia plugs were collected and placed for 60 min at 55 °C in an electric furnace to remove the water. A Lac part analytical balance was adopted to measure the wet weight of the dry mycelium. For comparisons of spore sporulation, 3 days of growth of the wild strain and positive transformant were cut into small pieces (2 mm × 2 mm) with a sterile knife. After incubation in MBM liquid medium in a shaking bath at a speed of 160 rpm and 28 °C for 5 days, we estimated the number of conidium using a hemocytometer. The mycelial plugs of the wild strain and positive transformant of three days of growth were prepared by using a punch with a diameter of 0.5 cm to detect the response to various stressors (hyperosmotic and oxidative stress and cell wall antagonists). The wild strain and transformant were cultured on CM medium that contained different concentrations of NaCl (1 M, 2 M), SDS (0.005%, 0.02%), Congo Red (CR) (0.2 mg/mL, 0.4 mg/mL) and H_2_O_2_ (2 mM, 4 mM). All assays used 3 independent replicates, and all data were analyzed by one-way ANOVA and Duncan’s range test in IBM SPSS Statistics 26 to measure specific differences between pairs of means. A *p*-value of <0.05 was considered statistically significant.

To analyze the germination of spores within the initial 8 h of the wild strain and the transformant, we used an electron microscope and a fluorescence microscope to observe the state of conidia, which were harvested from MBM liquid media cultured for 5 days at 0 h, 2 h, 4 h, 6 h and 8 h. To verify whether pathogenicity changed between the wild and transformant strains, the wild strain and transformant strains were inoculated on *Dianthus chinensis* detached leaves with uniform growth for 3 days. Each asymptomatic leaf was wounded at the one-third of the top of the leaves by using a sterile inoculation needle and we prepared the mycelial plugs by punching holes at the edge of the colony with a 0.5 cm punch. Sterile agar blocks of same size as the experimental group were used for the control group. The blocks were inoculated on *D. chinensis* detached leaves at the wound site. Then, the leaves were positioned on wet Petri dishes at 25 °C and sprayed once every 24 h to keep them moist, using three independent replicates to test the pathogenicity. The disease incidence was examined 7 days after inoculation.

## 3. Results

### 3.1. Screening of Resistance Concentration of FDCY-5 to Hyg

**Figure 1:**
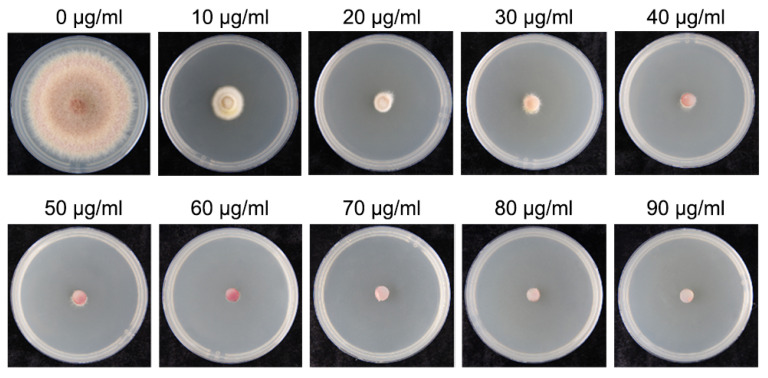
The inhibition of the different concentrations of Hyg antibiotic on mycelial growth of the wild-type FDCY-5 strain. The concentration (10, 20, 30, 40, 50, 60, 70, 80, or 90 μg/mL) of antibiotic is marked above the image.

The FDCY-5 grew with no inhibition on CM plates without Hyg antibiotics. However, its growth was significantly inhibited on CM plates containing Hyg at concentrations of 10, 20, 30, 40, 50, 60, 70, 80, and 90 μg/mL. Notably, mycelial growth was completely suppressed at a concentration of 60 μg/mL Hyg. This finding indicates that the minimum inhibitory concentration (MIC) of Hyg is established at 60 μg/mL and serves as the screening concentration for transformants (Figure 1). To ensure experimental accuracy in subsequent tests, a higher concentration of 100 μg/mL Hyg was utilized.

### 3.2. Effective Enzymatic Hydrolysis Time of Preparation of Protoplasts 

**Figure 2:**
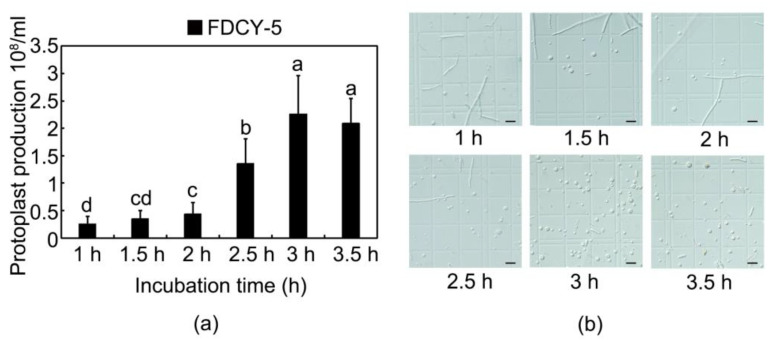
(**a**) Effect of enzymatic hydrolysis time on preparation of protoplasts; (**b**) Protoplast morphology at different enzymatic hydrolysis times. Bars, 20 μm; different lowercase letters in the same column show the significant differences in the quantity of protoplasts (*p* ≤ 0.05).

To enhance transformation efficiency, a critical factor was the hydrolysis duration of mycelium to achieve an adequate yield of protoplasts. As illustrated in Figure 2, extending the enzyme solution time resulted in a continuous increase in the number of protoplasts up to 180 min, followed by a slight decline at 210 min. At 180 min, the quantity of protoplasts reached 2.26 × 10^8^. Although there was no statistically significant difference in protoplast quality between the durations of 180 and 210 min, for optimal speed and efficiency, a hydrolysis time of 180 min at 30 °C with shaking at 80 rpm is recommended for preparing high-quality and maximum-yielding protoplasts (Figure 2). 

### 3.3. Transformation of F. acuminatum with GFP Gene

**Figure 3:**
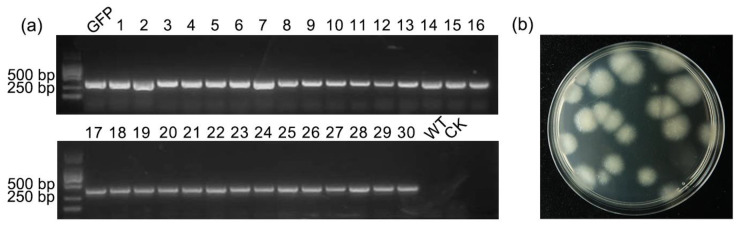
(**a**) Identification of *F. acuminatum* GFP transformants. GFP: PKD1-GFP vector; WT: FDCY-5 strain; CK: ddH_2_O; 1–30: FaGFP 1–30. (**b**) Obtained transformants in selective medium.

**Figure 4:**
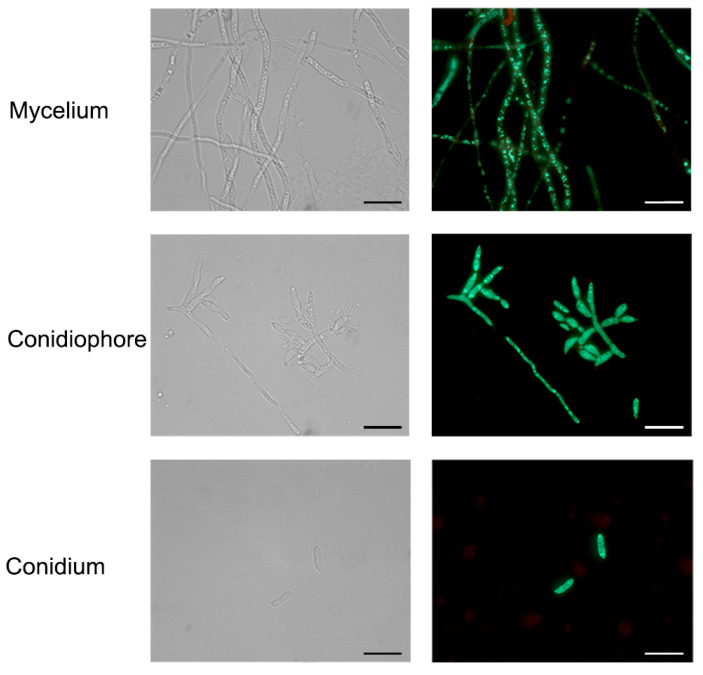
Mycelium, conidiophore and conidium (microspore) fluorescence observations of *F. acuminatum* transformants. Bars, 20 μm.

According to the protoplast transformation method established above, a total of 32 transformants were obtained, of which 30 exhibited amplified GFP bands (Figure 3a,b). In contrast, no DNA bands were detected in the wild strain or the empty vector. The PCR results indicated that the GFP gene was successfully transferred into the transformants. The amplification efficiency can reach up to 93.75%. All putative transformants were inoculate onto CM culture medium and cultured for three days to verify the expression of the GFP gene. The putative transformants exhibited green fluorescence, while the wild strain showed no fluorescence under fluorescence microscopy. These results indicated that the transformants containing the GFP gene successfully expressed GFP, further validating that the protoplast transformation method established above is effective. One isolate, FaGFP-1, was selected for further observation of GFP gene expression in mycelia and conidia. As illustrated in Figure 4, all conidia and sporulation displayed strong green fluorescence under fluorescence microscopy. The GFP protein was highly expressed in both the cytoplasm and nucleus of fungal cells as well as in conidia after three generations cultivation on selective plates containing 100 μg/mL Hyg, confirming the successful and stable expression of GFP (Figure 4).

### 3.4. Phenotypic Results of the Wild FDCY-5 Strain and FaGFP-1 

**Figure 5:**
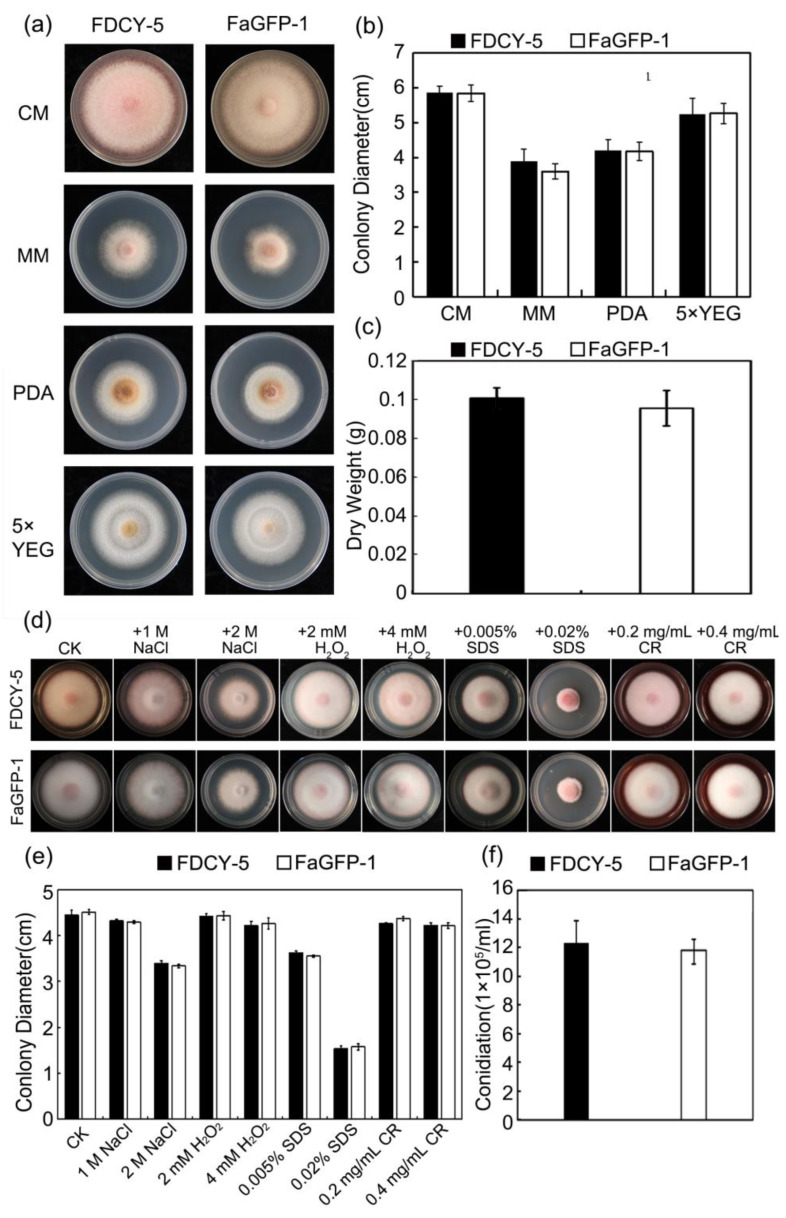
Phenotypic identification of wild strain (FDCY-5) and representative transformant (FaGFP-1). (**a**) Colony morphology of FDCY-5 and FaGFP-1 on various media (CM, MM, PDA, 5 × YEG) for 5 days; (**b**) Colony diameter of the two strains in different media; (**c**) Dry weight of FDCY-5 and FaGFP-1 after culturing for 16 h; (**d**) The response to various stressors of the two strains; (**e**) Colony diameter of the two strains on a CM plate containing different stressors; (**f**) Quantification of the conidia produced by FDCY-5 and FaGFP-1 (*p* ≤ 0.01).

The isolate FaGFP-1, exhibiting the strongest fluorescence, was selected for experimental evaluation. We assessed the growth of both FaGFP-1 and the wild type on four different medium plates: CM, MM, PDA and 5 × YEG. As illustrated in Figure 5a, colonies grew most rapidly and achieved the largest diameter on the CM plate within five days. In contrast, after five days of cultivation, both strains exhibited their slowest growth and smallest colony diameters on the MM plate. The isolate FaGFP-1 demonstrated no significant differences in vegetative growth, aerial hyphae formation, or pigment deposition when compared to the wild type (Figure 5a,b). Furthermore, regarding dry weight measurements after 16 h of culture in CM liquid medium (Figure 5c), no significant differences were observed between the two strains.

We also examined how both strains responded to various environmental stressors. No substantial inhibition was noted on plates containing 1 M NaCl or concentrations of CR at 0.2 mg/mL and 0.4 mg/mL. Similarly, there was no difference for H_2_O_2_ at concentrations of 2 mM and 4 mM when compared to control plates without stressors. However, a marked decrease in growth was observed under conditions with 2 M NaCl as well as with SDS concentrations of 0.005% and particularly at 0.02%. The growth rate of GFP-transformants showed no significant difference from that of the wild strain (Figure 5d,e). Furthermore, we observed the conidiation of the GFP-expressing isolate. After culturing in liquid medium for five days, conidial suspensions were collected for analysis. No significant differences in conidium production were noted when compared to the wild type (Figure 5f).

**Figure 6:**
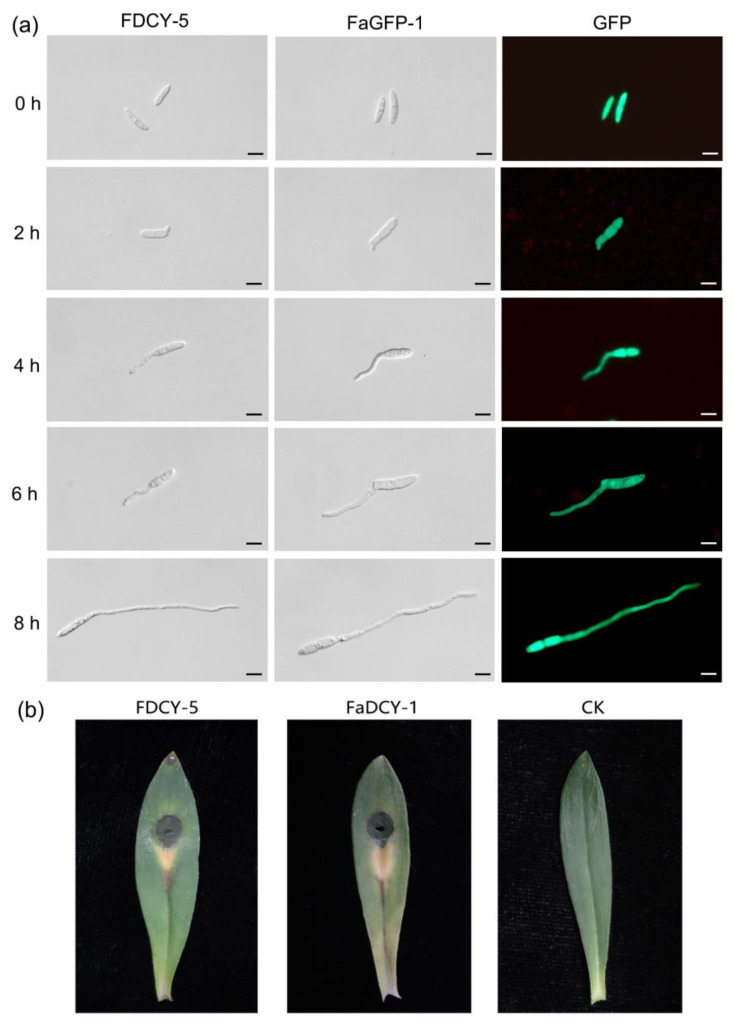
(**a**) Conidial germination of FDCY-5 and FaGFP-1 strains in liquid MBM at different times. Examination was conducted at 0, 2, 4, 6, and 8 h. Bar = 20 μm; (**b**) Pathogenicity of FDCY-5 and FaGFP-1 on *D. chinensis* leaves.

In addition, we assessed the germination of conidia from both strains. The mycelium of each strain was inoculated into liquid MBM medium for five days, after which conidia were harvested for germination testing. Samples containing spore suspensions at various time points were examined under a 40× microscope; the germination rates of both strains were found to be similar. Within 2–8 h post-inoculation, the tips of the mycelium continued to elongate. The elongated mycelium developed some septal hyphae and very few branches within this timeframe. These results indicate that there are no significant differences in growth, conidiation, or conidium germination between FaGFP-1 and the wild type, suggesting that the transformation with GFP did not affect the fundamental physiological characteristics of *F. acuminatum* (Figure 6a).

*F. acuminatum* has been proven to infect *D. chinensis,* causing symptoms of foliage blight and root discoloration on approximately one-year old *D. chinensis* plants. The pathogen can disseminate in soil to infect *D. chinensis* plant roots. Alternatively, it can rely on conidium to disseminate through the air and infect host plants. Typically, mycelium and conidium infect through wounds [20]. To determine whether FaGFP-1 influenced infection capabilities, we evaluated its colonization on leaves. Following incubation at 25 °C for five days, water-soaked lesions and yellowing symptoms were observed on leaves infected by both wild-type and FaGFP-1 strains (Figure 6b). The absence of significant differences in symptom severity between these two strains further indicates that protoplasts introduced with the GFP gene had no impact on pathogenicity.

## 4. Discussion

*Fusarium* spp., a genus within the Pezizomycotina, includes several phytopathogenic species such as *F. graminearum*, *F. oxysporum*, *F. proliferatum* and *F. verticillioides*. These fungi are responsible for diseases like *Fusarium* head blight and *Fusarium* wilt, which significantly impact global crop production [44,45,46]. Most *Fusarium* species are soil-borne pathogens that spread through soil [47] and can interact with the host microbiome at various stages of their life cycles and across different plant organs, thereby influencing host growth [48]. Asexual spores play a critical role in the propagation and infection processes of *Fusarium* [49]. *F. acuminatum* is widely distributed across the globe, including regions such as Canada, Greece, the United States, South Africa, and China [50,51,52]. Increasing reports have emerged regarding the detrimental effects of *F. acuminatum* in China, leading to significant plant wilting or mortality. Therefore, there is an urgent need for reasonable and effective disease control measures to be implemented.

At present, fungicides are primarily employed to manage diseases caused by *Fusarium* species. However, these pathogenic fungi exhibit a propensity for developing drug resistance, which diminishes the effectiveness of these fungicides. Therefore, it is essential to explore additional drug targets as a foundation for the development of novel fungicides. 

To more effectively identify the pathogenic target genes of *F. acuminatum*, establishing a high-efficiency transformation system should be prioritized. A commonly employed method for fungal transformations is *Agrobacterium*-mediated transformation (ATMT). Compare to the PMT method, ATMT offers several advantages, including a wider range of transformation recipients—such as protoplasts, hyphae, and spores—and higher transformation efficiency [26]. *Aspergillus flavus* transformants were successfully generated with an efficiency of 60 positive transformants per 10^6^ conidia [53]. The ATMT system of *A. awamori* obtained 200–250 transformants per 10^6^ conidiospores [54]. In terms of experimental operation, our PMT system is more straightforward compared to ATMT; preparing binary vectors for ATMT can be labor-intensive and its overall transformation process tends to be more complex. Given the high efficiency associated with the protoplast enzyme systems we have established, our PMT system has proven to be more suitable for practical experimentation.

In this research, we initially established an efficient and stable method for the transformation of *F. acuminatum* based on several PEG-mediated protoplast transformation techniques [30,55,56]. A substantial quantity of protoplasts is essential for a successful and effective protoplast-mediated transformation (PMT) system. In *Valsa mali*, the number of protoplasts can reach 4 × 10^7^ in 1 mL STC after incubation in enzymatic hydrolysate containing 50 g/L Driselase and 10 g/L lysing enzymes. For *Cytospora chrysosperma*, 1 × 10^8^ protoplasts were collected in 1 mL STC following 4 h of enzymatic hydrolysis with a solution comprising 20 g/L Driselase and 8 g/L lysing enzymes [57]. For *F. graminearum,* 3.4 × 10^5^ protoplasts were obtained in 10 mL of enzyme solution for 3 h [58]. In comparison to these transformation systems, our findings indicate that after three hours of enzymatic hydrolysis, the concentration of protoplasts reached 2.26 × 10^8^/mL. Our system demonstrates significant advantages regarding enzymatic hydrolysis duration, yield of released protoplasts, as well as both the quantity and cost-effectiveness of the polymerase used. Furthermore, our investigation successfully identified thirty transformants that were effectively transduced with the GFP gene and demonstrated high levels of GFP protein expression.

The adoption of the PEG-mediated protoplast transformation method offers advantages such as simplicity, convenience, and high efficiency, making it particularly suitable for *F. acuminatum* [59]. In this study, we successfully obtained transformants expressing the GFP gene using the PEG (polyethylene glycol)/CaCl_2_ mediated protoplast transformation method. We observed several biological characteristics of the *F. acuminatum* GFP-transformants while they continuously grew to the third generation on CM plates. Subsequently, we compared growth rate, dry weight, spore production, responses to various stressors, spore germination, and pathogenicity between the transformant FaGFP-1 and wild type. Our findings indicated no significant differences between them. This suggests that the introduction of GFP did not affect the fundamental physiological traits or pathogenicity of *F. acuminatum*. The protocol we developed has proven to be effective and reproducible. When combined with gene knockout technology, it enables rapid identification of key genes related to pathogenicity in *F. acuminatum*, potentially providing new targets for pesticide development.

## Data Availability

All data generated or analyzed during this study are included in this article.
